# Efficiency of recombinant Ybgf in a double antigen-ELISA for the detection of Coxiella antibodies in ruminants

**DOI:** 10.1016/j.vas.2024.100366

**Published:** 2024-06-02

**Authors:** Gianmarco Ferrara, Barbara Colitti, Flores-Ramires Gabriela, Sergio Rosati, Giuseppe Iovane, Ugo Pagnini, Serena Montagnaro

**Affiliations:** a Department of Veterinary Medicine and Animal Productions, University of Naples, “Federico II”, Naples 80137, Italy; b Department of Veterinary Science, University of Turin, Grugliasco, TO 10095, Italy; c Institute of Virology Slovak Academy of Sciences, Bratislava 84505, Slovakia

**Keywords:** Ybgf, recombinant ELISA, double-antigen ELISA, Q fever, Coxiella, Ruminants

## Abstract

Q fever is a zoonosis whose main reservoirs are domestic ruminants. Surveillance in these species is carried out mainly with serological tests, which, however, have limited diagnostic performance, and their manufacturing requires laboratories equipped with high biosafety requirements for antigen production. Recombinant ELISAs do not depend on these requirements and, being based on a single antigen, can reduce potential false positivity by identifying antibodies specific to a phase of infection. The aim of this study was to apply a new technology (dual antigen test) to a recombinant protein (Ybgf), an antigen produced in recombinant form and already used in previous studies for the design of an indirect ELISA. The successfully produced recombinant antigen was used to coat 96-well plates and, at the same time, another antigen aliquot was conjugated with HRP to obtain an HRP-conjugated Ybgf. After setting the test conditions, the results obtained with the recombinant double antigen test were compared with those obtained with a commercial assay (considered as reference assay) testing a total of 514 ruminant samples (280 goats and 234 cattle). A concordance of 86.2 and a Cohen's Kappa value of 0.72 were obtained, with no significant difference between the two species tested. Notably, the test proved to be highly specific, having correctly identified 250 out of 253 animals. This research represents an additional effort to use recombinant antigens to enhance serological methods in veterinary medicine. In a “one-health scenario”, improving the performance of serological tests used in veterinary practice also means improving the surveillance of this infection.

## Highlights

• Recombinant ELISAs could solve some problems observed in Q fever serology.
• Double-antigen YBGF revealed good concordance with a commercial assay.
• The production of double-antigen YBGF was cheaper and simpler.

## 1 Introduction

Q fever is a zoonosis responsible for public health problems as well as economic losses on livestock farms (mainly for ruminants) (Angelakis & Raoult, 2010; Eldin et al., 2017). *Coxiella burnetti*, (*C. burnetii*) a Gram-negative bacterium that is mainly shed through aborted fetuses and vaginal secretions, causes this infection. Furthermore, the bacterium could be shed during live births from apparently healthy animals. Contagion occurs both through the respiratory route (more common) and through ingestion of contaminated food, both in animals and humans (Angelakis & Raoult, 2010; Guatteo et al., 2011). *C. burnetii* is currently widespread across all continents, and outbreaks are continuously reported in susceptible species (Eldin et al., 2017; Ferrara et al., 2022a). One of the main problems in the surveillance and management of this infection is the diagnostic performance of the serological tests used to identify exposed animals (Paul et al., 2013). Complement fixation test (CFT) was considered the serological test of choice in the past, although it was later replaced by the enzyme-linked immunosorbent assay (ELISA) (Sahu et al., 2020; Szymańska-Czerwińska et al., 2016). These tests have the advantage of being relatively rapid, inexpensive, and easily applicable for a herd survey (Horigan et al., 2011). An immunofluorescence assay (IFA), considered the gold standard test in humans, has recently been validated for ruminants, but the need to test and interpret many contemporary samples limits its use on a large scale to the advantage of ELISA, which has been suggested by the WOAH (World Organisation for Animal Health) guidelines as a screening assay (Lucchese et al., 2015; Wood et al., 2019). The commercial tests currently available are based on the corpuscular antigens of *C. burnetii* reference strains. The production of these antigens is time-consuming and expensive, as well as requiring a biosafety level 3 laboratory (BSL3) (Sahu et al., 2020). On the other hand, the diagnostic performances (sensitivity and specificity) could be influenced by the variance of *C. burnetii* strains and genotypes circulating in different territories, by the antigenic homology between *C. burnetii* and other pathogens (described for *Chlamydia* spp., *Rickettsia* spp., and *Bartonella* spp.), by the species, and by the matrix (Emery et al., 2012; Lurier et al., 2021; Paul et al., 2013). Furthermore, due to the nature of the antigen, these assays cannot distinguish whether the antibodies are directed toward phase II (as in acute forms) or towards both phases of *C. burnetii* (as in chronic forms) (Bauer et al., 2016).

In recent years, good results have been obtained with the use of recombinant antigens, which are less expensive, less laborious to produce (BLS3 is not required), and specific to an infection phase (Miller & Kersh, 2020). When key immunodominant *C. burnetii* antigens were used for recombinant ELISAs and compared to commercial ELISAs, several studies revealed significant concordance rates. Ybgf (CBU_0092), also known as cell division coordinator CpoB and involved in the Tol-Pal transport system and synthesis of peptidoglycan, is one of the most investigated antigens for its potential use in human and animal vaccinology and diagnostics (Dold et al., 2023; Vigil et al., 2010; Xiong et al., 2012). Several studies that have identified Ybgf as an early marker of infection have established that the anti-Coxiella humoral response is localized to this periplasmic protein Beare et al. (2008); Deringer et al. (2011); Gerlach et al. (2017). This antigen was recently employed in the development of indirect recombinant ELISAs for detecting anti-Coxiella antibodies in ruminants (Ferrara et al., 2022b). In some cases, the use of double antigen ELISA technology (using the same antigen coated on the plate as secondary HRP-conjugated) allows for improved diagnostic performances. Given the promising results obtained in a previous study, the aim of this study was to use this antigen to develop a double-antigen ELISA for the detection of *C. burnetii* antibodies in ruminants.

## 2 Materials and methods

### 2.1 Production of recombinant YBGF in *Escherichia coli*

*C. burnetii* Nine Mile RSA 493 phase I strain was grown in axenic media in a BSL3 facility as previously described (Zuñiga-Navarrete et al., 2019). After centrifugation of the bacteria at 15,000x*g* for 1 hour at 4 °C, DNA was extracted (DNeasy blood and tissue kit; Qiagen) according to the manufacturer's recommendations. According to the Ybgf gene (Q83F57) deposited in NCBI (https://www.ncbi.nlm.nih.gov/), specific primers including *Bam*HI and EcoRI restriction enzyme sites (underlined) were constructed, excluding the leader peptide (Forward 5′ TTGGATCCCCAGTGGAAGATATTTCGGCG CAAC-3′; Reverse 5′-TTGAATTCAGGCGTT GTTGTTGCTGAATCGAC-3). A total of 34.3 µL of RNase-free water, 5 µL of CoralLoad buffer 10 (Qiagen), 1.5 µL of MgCl2 (50 mM), 1 µL of dNTPs (10 mM), 2.5 µL of each primer (10 M), 3 µL of gDNA (previously measured at 50 ng/µL), and 0.25 µL of Taq DNA polymerase (Qiagen) were used to generate an 822-bp product (Ferrara et al., 2022b). PCR cycling conditions were described as follows: 3 min of PCR activation at 94 °C, 45 s of denaturation at 94 °C, 35 cycles of annealing at 60 °C for 30 s, and elongation at 72 °C for 90 s, followed by a final elongation at 72 °C for 10 min. The 882-bp product was evaluated on a 1.5 % agarose gel, digested with *Bam*HI and EcoRI (Thermo Scientific) for 3 h at 37 °C, purified, and ligated into a pGEX-6P expression vector (previously digested with the same enzymes). The ligation product was used to transform competent *E. coli* BL21 C43 (DE3) cells plated in Luria-Bertani (LB) medium. Positive colonies were grown in LB supplemented with ampicillin and stimulated with 1 mM isopropyl-d-1-thiogalactopyranoside (IPTG) during the mid-exponential phase, following confirmation by tapping in colony PCR. Sanger sequencing of the DNA plasmid generated from a positive clone provided additional confirmation of the insert's features and in-frame orientation. Bacterial pellets were lysed with lysozyme, sonicated, and then purified under native conditions (using an on-column sepharose resin in three affinity chromatography steps) (glutathione Sepharose 4B resin; Merck) (Cavalera et al., 2021). To ensure that the fusion carrier (GST) did not interfere with the expected reactivity, cleavage was done directly on columns using PreScission protease (2 U/mg; Merck). SDS-PAGE (stained with Coomassie brilliant blue R250) was utilized to separate the proteins into each eluate and determine the correct size and amount. The identity of the protein was confirmed in previous work using mass spectrometry (Ferrara et al., 2022b).

### 2.2 Generation of HRP-conjugated recombinant YBGF

Conjugation of recombinant Ybgf with HRP was performed by hydrating a vial of HRP (Merck) in 450 µl of water. A total of 120 µl of 0.088 M sodium periodate solution was added and incubated for 15 min in the dark. At the end of the incubation (the product had turned green), the sodium periodate was inactivated by adding 60 µl of ethyl glycol (the product had turned brown again). A total of 3 mg of Ybgf antigen dialyzed against carbonate buffer at pH 9.6 were added to the solution and incubated for two hours at room temperature in the dark. After the addition of 250 µl of sodium boron hydride, a further two-hour incubation at 4 °C was carried out (opening the container cap every hour to reduce the internal pressure due to the release of oxygen). The final product was dialyzed overnight at 4 °C against 100 vol of Tris 20 mM pH 7.5 and 0.5 M NaCl. The entire volume was purified by affinity chromatography with one ml of resin ConA-SepharoseB (Merck), incubated for 30 min on a shaker. The eluations (4) were carried out with 1 M sucrose (4 vol of Guardian diluent were added to each elution) and stored in the refrigerator until use. After confirmation of conjugation (by Coomassie-stained SDS and ELISA), the eluates were mixed and diluted in HRP stabilizer.

### 2.3 Development of the double-Ybgf Elisa

The double-antigen assay is based on the coating of small quantities of antigen, which are incubated with poorly diluted serum samples and, subsequently, with the secondary antigen (conjugated to HRP). The recombinant protein was quantified (using the Bradford protein assay, Thermo Fisher), diluted to 0.25 ng/µL in 0.1 M carbonate-bicarbonate buffer (pH 9.6), and used to coat (100 µL) the wells of 96-well plates overnight at 4 °C (Nunc Maxisorp; MilliporeSigma). After blocking with 2.5 % bovine casein (300 µL), serum samples diluted 1:4 in PBS-1.25 % casein (100 µL) were added to the wells and incubated for 45 min at room temperature. Following a wash step, a 1:2 HRP-conjugated Ybgf was incubated in each well for 30 min at room temperature. After a further wash step, the reaction was developed with 100 μL of 3,3′,5,5′-tetramethylbenzidine (TMB) and stopped with 100 μL of 0.2 M H2 SO4. Optical density (OD) was measured at 450 nm. To distinguish between positive and negative samples, a cutoff value of 0.3 was arbitrarily chosen (average OD of negative sera+4 SDs). These settings were chosen by testing different amounts of coated antigen (8, 25, and 50 ng), HRP-conjugated Ybgf (not diluted, 1:2 and 1:4), and serum dilutions (1:2, 1:4, 1:8) of positive and negative control sera (crisscross serial-dilution of an initial panel of sera consisting of 6 negative and 6 positive cattle) (Ferrara et al., 2023a). A panel of apparently healthy ruminant sera belonging to other studies (280 goats and 234 cattle) was randomly chosen to investigate the potential of double-Ybgf ELISA against a commercial one (Q fever Ab test kit; Idexx) (Ferrara et al., 2022a, 2023b, 2023c). The commercial indirect ELISA was performed following the manufacturer's instructions. Briefly, 1:100 diluted sera were incubated for one hour in an ELISA plate. After three washing steps, 100 µL of secondary HRP-conjugated was provided for another hour. Further three washing steps preceded the colorimetric reaction with TMB and stop solution. The OD was measured at 450 nm.

### 2.4 Statistical analysis

The agreement between the results obtained with double-Ybgf and those obtained with the commercial ELISA was calculated. The Cohen κ coefficient was used to determine the level of agreement between the 2 tests (0.81–1.00 = almost perfect agreement; 0.61–0.80 = substantial agreement; 0.41–0.60 = moderate agreement; 0.21–0.40 = fair agreement; 0.01–0.20 = slight agreement; 0.00 = no agreement). Statistical analysis was performed with MedCalc v.18.11.3 (MedCalc Software) while GraphPad Prism 8 software (GraphPad Prism) was used to create Fig. 2, Fig. 3. Kruskal-Wallis tests were used to assess the results concerning the assay validation steps. Significant differences were determined at *p* < 0.05 (GraphPad Prism).

## 3 Results

**Fig. 1:**
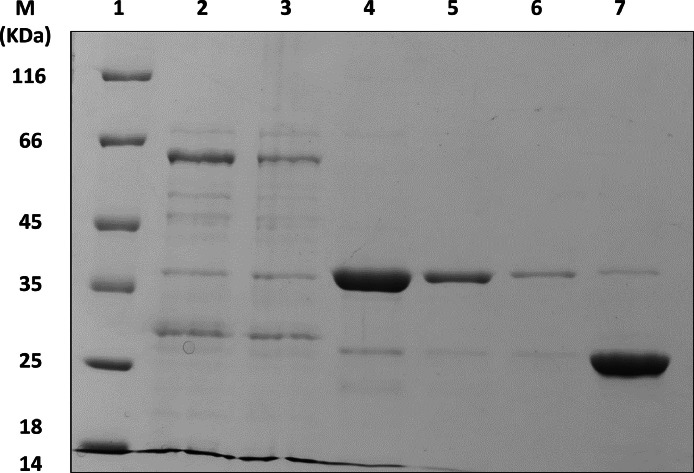
SDS-PAGE stained with Coomassie of recombinant Ybgf. Line 1: Molecular weight (as indicated by the manufacturer). Lane 2: Total extract. Lane 3: Total extract after resin adsorption. Lane 4, 5, 6: Purified and cleaved Ybgf after chromatography steps combined with protease treatment. Lane 7: Resin. In lanes 2 and 3, a main band of approximately 65 KDa is highlighted (35 KDa for Ybgf and approximately 20 for GST). The band reduces in intensity after adsorption with the resin. In lanes 4, 5, and 6, the main band has a molecular weight of 35 KDa (GST has been eliminated). In lane 7 (resin), a band weighing approximately 20 KDa (GST) is highlighted.

**Fig. 2:**
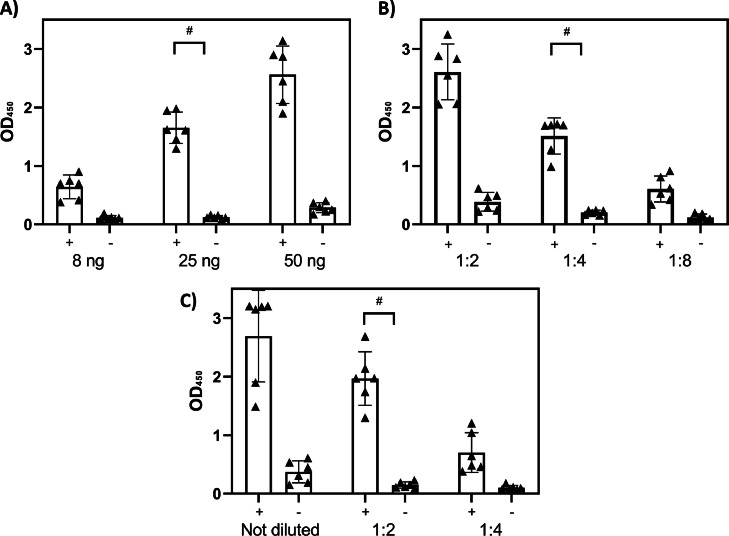
The serum dilution and antigen concentration that perform optimally for the double-Ybgf ELISA testing six positive and six negative serum samples. A) Using different amount of antigen (8, 25, and 50 ng), (B) using different serum concentrations (1:2, 1:4, 1:8), and (C) using different conjugated antigen amounts (not diluted, 1:2 and 1:4). Hashes indicate optimal conditions. The bars represent the x̄ ± standard deviation (error bars) of the OD of each antigen and serum dilution.

The recombinant Ybgf antigen was successfully produced, as indicated by Sanger sequencing of positive *E.coli* clones and as evidenced by the presence of a band with a molecular weight of approximately 65 KDa in Coomassie-stained SDS Page (Fig. 1). The fusion protein (Ybgf+GST) was found in the soluble fraction, as much of it was concentrated in the supernatant of the transformed *E.coli* cultures and did not require purification under denaturing conditions using urea. Through adsorption with a specific resin, the affinity between the GST and the sepharose (contained in the resin) was exploited, which allowed the purification of the antigen (Fig. 1). During the chromatographic steps, the use of a protease removed the GST from the fusion protein, releasing exclusively the Ybgf antigen (with a molecular weight of 35 KDa). However, much of the GST remained in the resin. Part of the antigen was used for coating 96-well plates, while a quantity equal to 3 mg was successfully conjugated to HRP and stabilized in a specific buffer. HRP-conjugated Ybgf was evaluated under different experimental conditions using different amounts of coated antigen in the plate, different dilutions of serum, and different dilutions of conjugated antigen (crisscross serial dilution analysis) (Fig. 2). These dilutions and concentrations showed a clear and quantifiable reaction (OD450 greater than 1) when reacting with positive sera and no colorimetric reaction in the presence of negative sera.

**Fig. 3:**
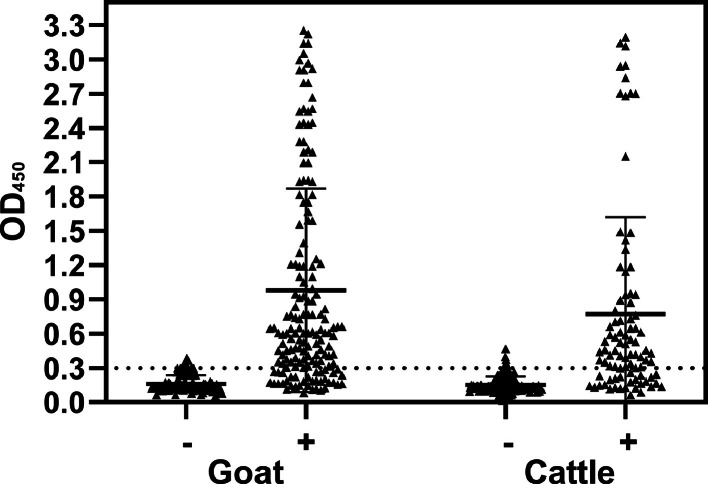
Double-Ybgf ELISA results testing 514 ruminants (280 goats and 234 cattle). The central bar and whiskers represent the OD's x̄ ± SD. The cut-off value is indicated by the dashed line.

Once the best conditions for discriminating seropositive animals have been identified (25 ng of coated antigen, serum dilution 1:4, HRP conjugated-antigen dilution 1:2), they were used to test 514 sera from ruminants (280 goats and 234 cattle) whose seroreactivity against *C. burnetii* had been assessed with a commercial indirect ELISA kit (Fig. 3). Discordant results were observed in both seropositive goats and cattle (32 out of 162 in the goat and 26 out of 89 in the bovine species) (Table 1). A total of 250 out of 263 animals negative for the commercial test was also negative for the double-Ybgf test (Table 1). A total concordance of 86.2 % was observed, with no significant differences between the two species involved (86.1 % in goats and 86.3 % in cattle) (Table 2). Consequently, Cohen's kappa values were also very similar between the two species (0.72 in goats and 0.69 in cattle) (Table 2).

**Table 1:** Results obtained with commercial ELISA and double-Ybgf testing 514 ruminant serum samples.

Species	+ IDEXX	- IDEXX	Total
Goat			
+ double-Ybgf	130	7	
- double-Ybgf	32	111	
Total	162	118	280
Bovine			
+ double-Ybgf	63	6	
- double-Ybgf	26	139	
Total	89	145	234
Overall			
+ double-Ybgf	193	13	
- double-Ybgf	58	250	
Total	251	263	514

**Table 2:** Agreement between commercial ELISA and double-Ybgf.

Species	Agreement	95 % CI	Cohen κ	95 % CI
Goat	86.1	81.45–89.9	0.72	0.64–0.8
Cattle	86.3	81.25–90.45	0.69	0.60–0.79
Overall	86.2	82.9–89.05	0.72	0.66–0.78

CI= Confidential Interval.

## 4 Discussion

Improving the characteristics of serological tests used in ruminants is a veterinary mission in Q fever prevention (Bauer et al., 2023a). In this study, a double-antigen ELISA using Ybgf was compared to a commercial kit for the first time, obtaining good results. The concordance values obtained are similar to those obtained in other studies that have used recombinant antigens to detect antibodies against *C. burnetii* in ruminants. There were no noticeable differences between goats and cows. Differences can appear as a result of different antibody responses to *C. burnetii* among species. For example, small ruminants exhibit a stronger reaction to phase I (due to numerous chronic infections) than other ruminant species (Emery et al. 2012). The same antigen (Ybgf) was used in an indirect ELISA, showing a sensitivity of 80 % and a specificity of 90 %, as well as a lack of reactivity in vaccinated animals (Ferrara et al., 2022b). Other antigens exploited for their immunological properties were SucB, HspB, and Com1 (the latter also used in a LAT assay) (Fernandes et al., 2009; Ferrara et al., 2023a; Stellfeld et al., 2020; Yadav et al., 2020). Except for HspB, which revealed higher performance even though it was tested on a smaller number of samples, the other antigens showed concordances of approximately 80 % (Fernandes et al., 2009). These assays showed a reduced sensitivity since they depend on a single antigen (typically specific to an infection phase), although they don't show several problems typical of commercial tests, such as cross-reactions with other pathogens and production difficulties (Ferrara et al., 2023a). However, the evidence reported in the scientific literature currently does not justify replacing commercial kits with recombinant ELISAs. On the other hand, the commercial test used for comparison performed poorly as evidenced in previous studies, which, in the absence of a gold standard and reference sera, raised concerns about some false-negative samples that could be false-positive for the commercial kit (Hogerwerf et al., 2014).

In particular, double-Ybgf correctly identified250 out of 263 seronegative animals and 193 out of 251 seropositive animals. This double antigen assay is based on the coating of small quantities of antigen (in order to not completely saturate the wells), which are incubated with poorly diluted serum samples and, subsequently, with an antigen conjugated to HRP. This type of assay, already commercially available for the detection of specific antibodies against *C. burnetii* but based on native antigens, has the advantage of being more specific, being able to also capture IgM, and, given the properties of the secondary antigen, could be considered suitable as a multispecies (Bauer et al., 2023b; Szymańska-Czerwińska et al., 2016). In fact, the double antigen assay identified a greater percentage of seronegative animals (250/263) than the indirect assay (390/433) based on the same antigen (Ybgf) and using the same indirect ELISA for the comparison (Ferrara et. al., 2022b).

Ybgf has been found to be one of the most important immunodominant antigens in humans and laboratory animals, as well as one of the most promising candidates for future applications in serodiagnosis and vaccinology (Bach et al., 2023; Jiao et al., 2014; Sam et al., 2023). This periplasmic protein is considered a specific marker of PhII (acute infection). This peculiarity, if also confirmed in ruminants, would explain the reduced performance of r-Ybgf and double-Ybgf in identifying seropositive animals (in chronically infected animals, reduced reactivity towards phase II in favor of that towards phase I has been described) (Serrano-Pérez et al., 2015). Because there was no information on the stage of infection in positive animals, identification failure in chronic infections was expected. Given the presence of commercially available ELISAs specific to a single phase of infection, it would be advisable to evaluate the performance of phase-specific antigens with the respective phase ELISAs (Bauer et al., 2023b). The number of false negatives should therefore be lower. The most promising antigens for each phase could therefore be selected, and a recombinant ELISA based on a mixture of these two antigens could be designed in order to detect antibodies against both phases of infection.

A disadvantage already reported for some recombinant antigens is related to the production in *E. coli* and its incapacity to carry out post-translational changes (such as glycosylation or methylation), which could contribute to the epitope definition (the antigen produced does not necessarily retain the antigenic characteristics of native protein). The use of innovative veterinary approaches for the identification of exposures is the basis for the prevention of this infection in reservoirs. In human medicine, for example, a chemiluminescent immunoassay with good performance when compared with ELISAs and IFA has recently been described (Gutiérrez-Bautista et al., 2024).

Considering these improvements and these limits, the applicability of recombinant tests for the detection of antibodies against *C. burnetii* in ruminants, although promising, requires further investigation (an ideal approach can only be pursued using reference sera obtained from experimental infections).

## Ethical statement

The Institutional Ethics Committee of the Department of Veterinary Medicine and Animal Production (Centro Servizi Veterinari), University of Naples, Federico II, authorized the animal study protocol (PG/2022/0093419) on July 20, 2022. Animals were handled in accordance with the guiding standards for biomedical research. Veterinarians obtained blood samples in accordance with good medical practices.

## Funding

This research was supported and funded by

## Ethical statement

Hereby, I, Gianmarco Ferrara, consciously assure that for the manuscript “Efficiency of recombinant Ybgf in a double antigen-ELISA for the detection of Coxiella antibodies in ruminants” the following is fulfilled:

1) This material is the authors' own original work, which has not been previously published elsewhere.
2) The paper is not currently being considered for publication elsewhere.
3) The paper reflects the authors' own research and analysis in a truthful and complete manner.
4) The paper properly credits the meaningful contributions of co-authors and co-researchers.
5) The results are appropriately placed in the context of prior and existing research.
6) All sources used are properly disclosed (correct citation). Literally copying of text must be indicated as such by using quotation marks and giving proper reference.
7) All authors have been personally and actively involved in substantial work leading to the paper, and will take public responsibility for its content.

## CRediT authorship contribution statement

**Gianmarco Ferrara:** Writing – review & editing, Writing – original draft, Software, Methodology, Investigation, Formal analysis, Conceptualization. **Barbara Colitti:** Writing – review & editing, Methodology, Investigation, Formal analysis, Conceptualization. **Flores-Ramires Gabriela:** . **Sergio Rosati:** Writing – review & editing, Supervision, Data curation, Conceptualization. **Giuseppe Iovane:** Visualization, Validation, Supervision, Project administration. **Ugo Pagnini:** Writing – review & editing, Visualization, Supervision, Project administration, Conceptualization. **Serena Montagnaro:** Writing – review & editing, Supervision, Software, Formal analysis.

## Declaration of competing interest

The authors declare that they have no known competing financial interests or personal relationships that could have appeared to influence the work reported in this paper.
